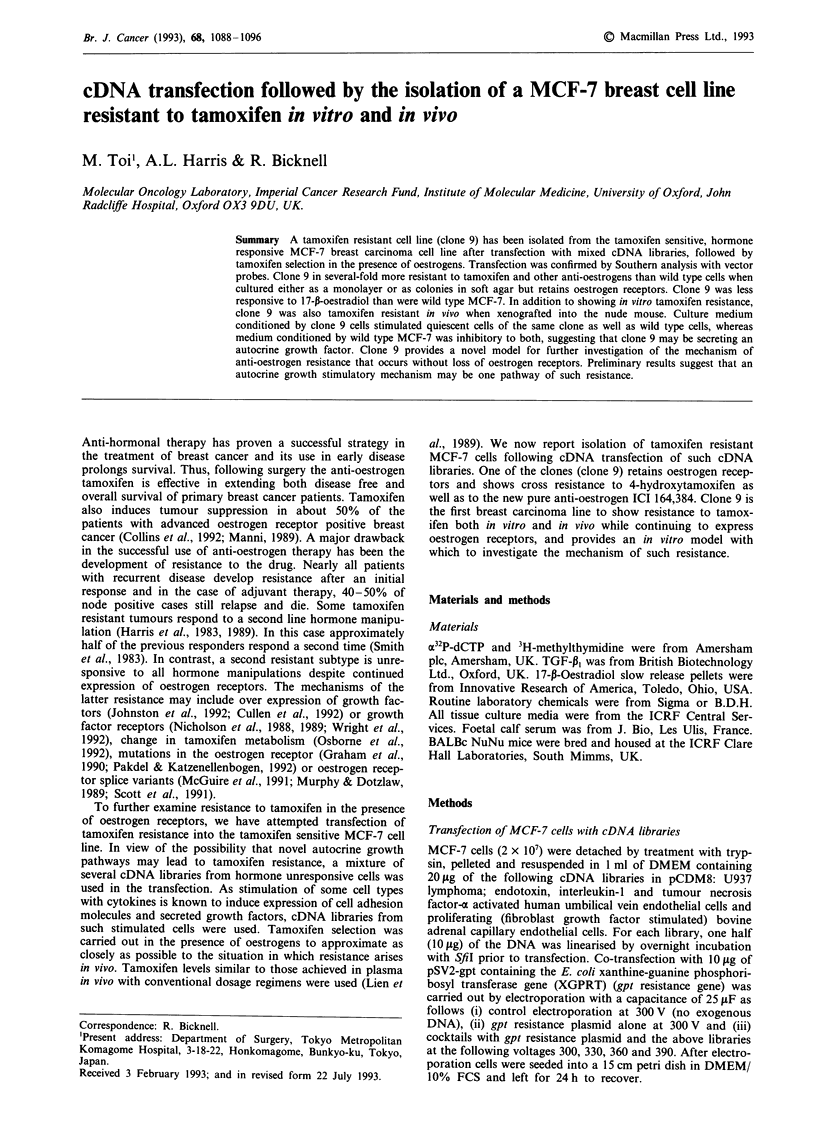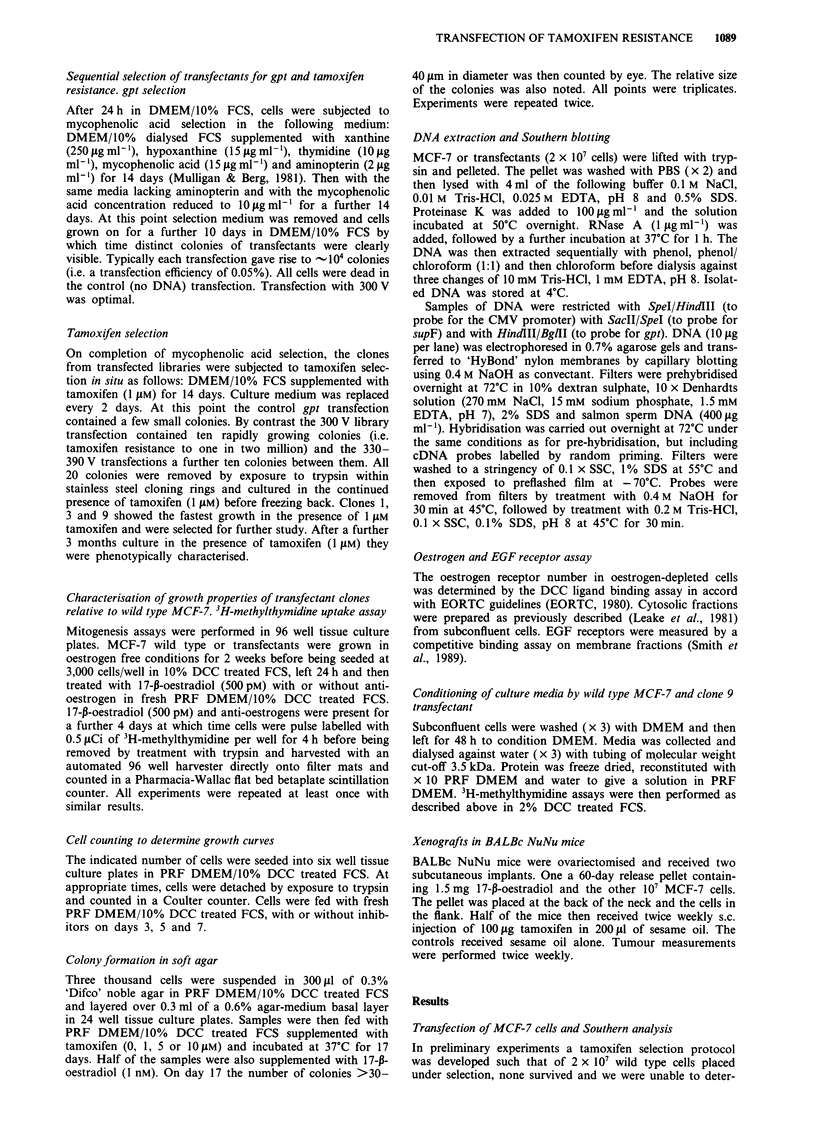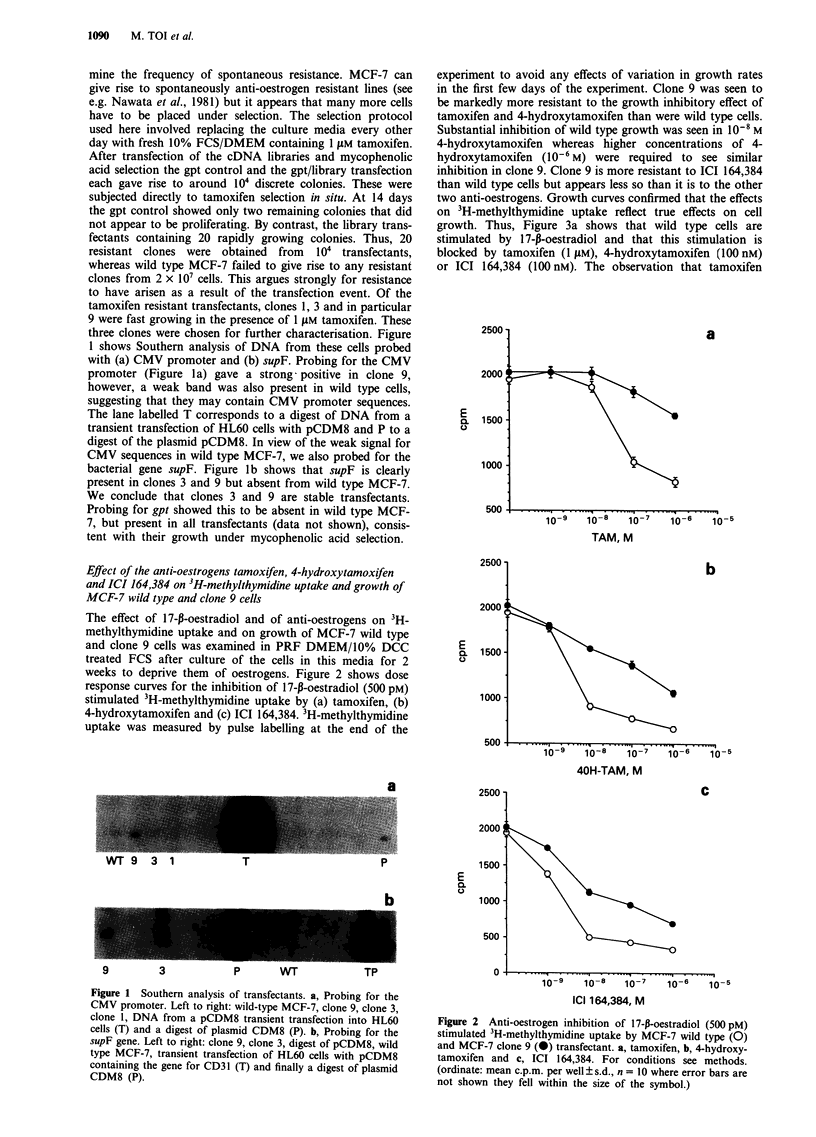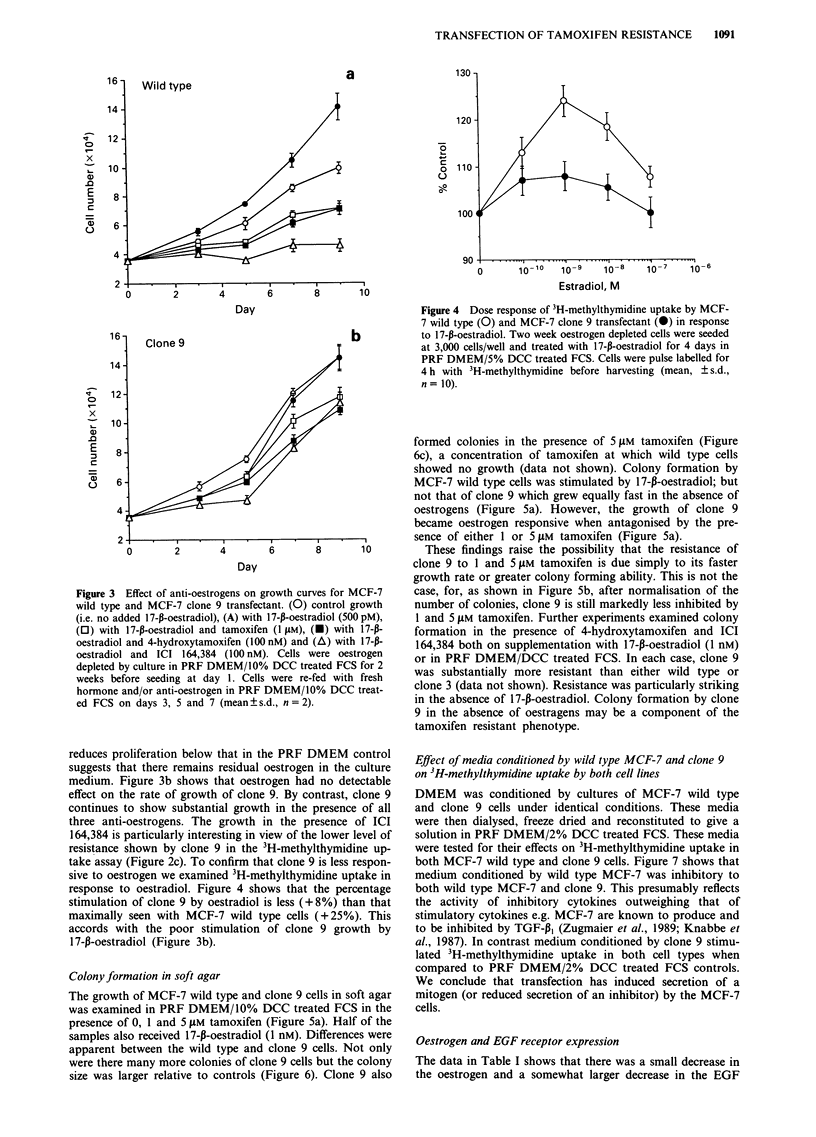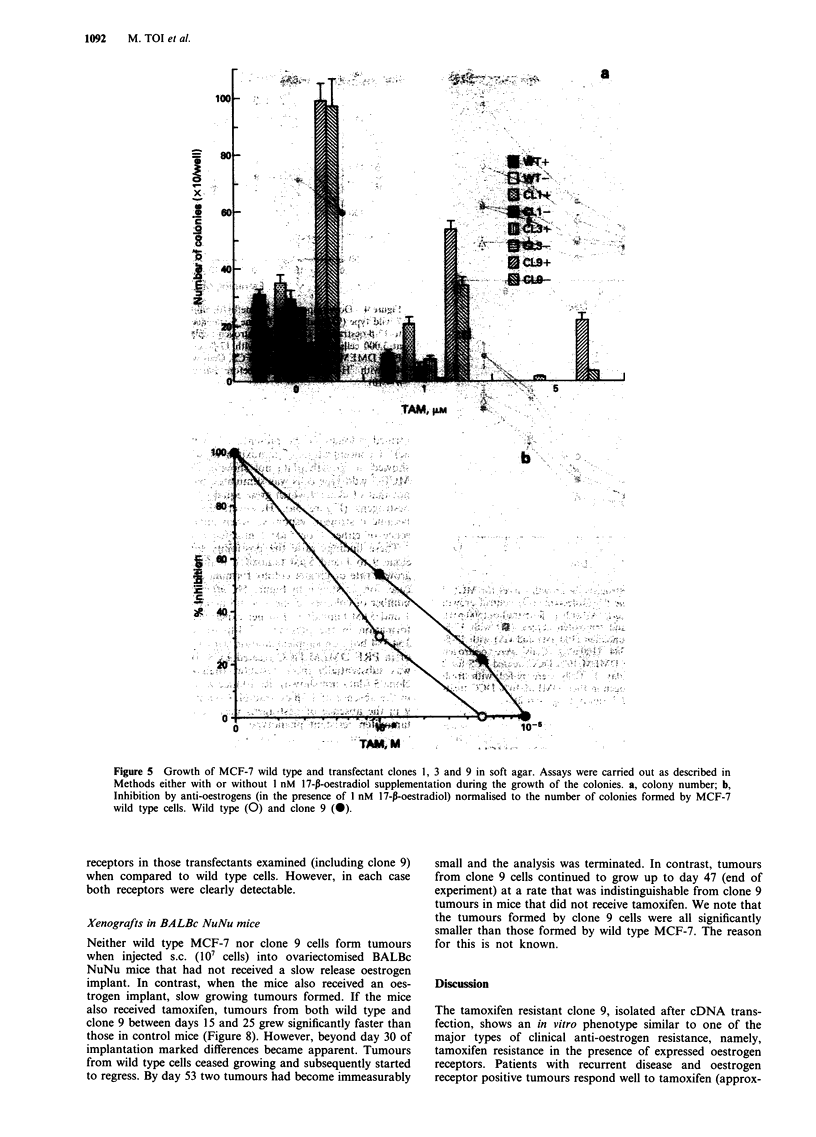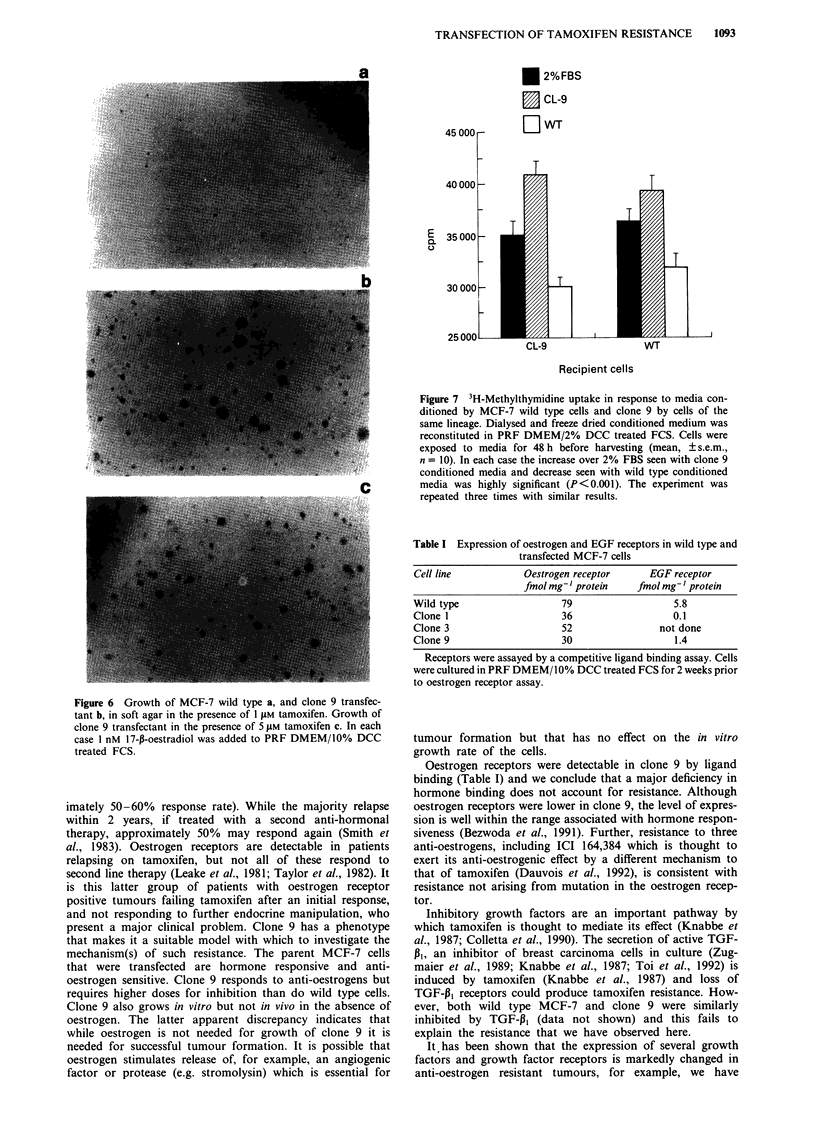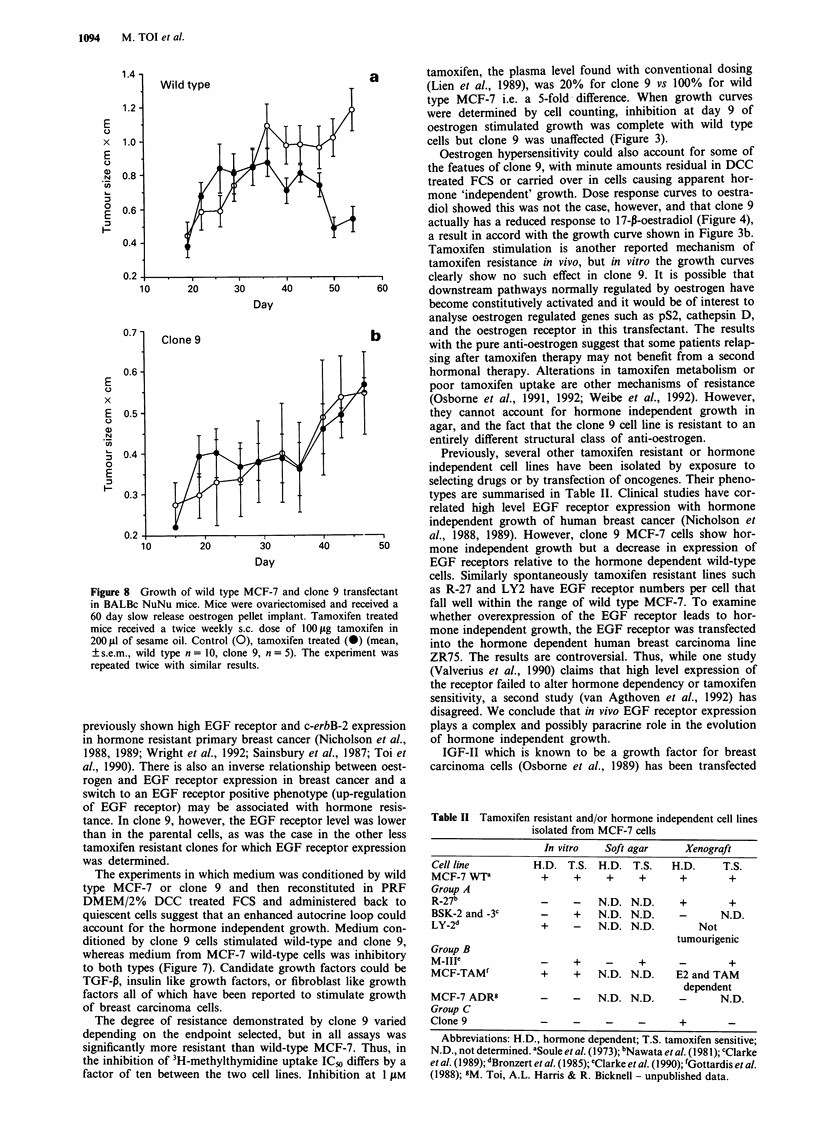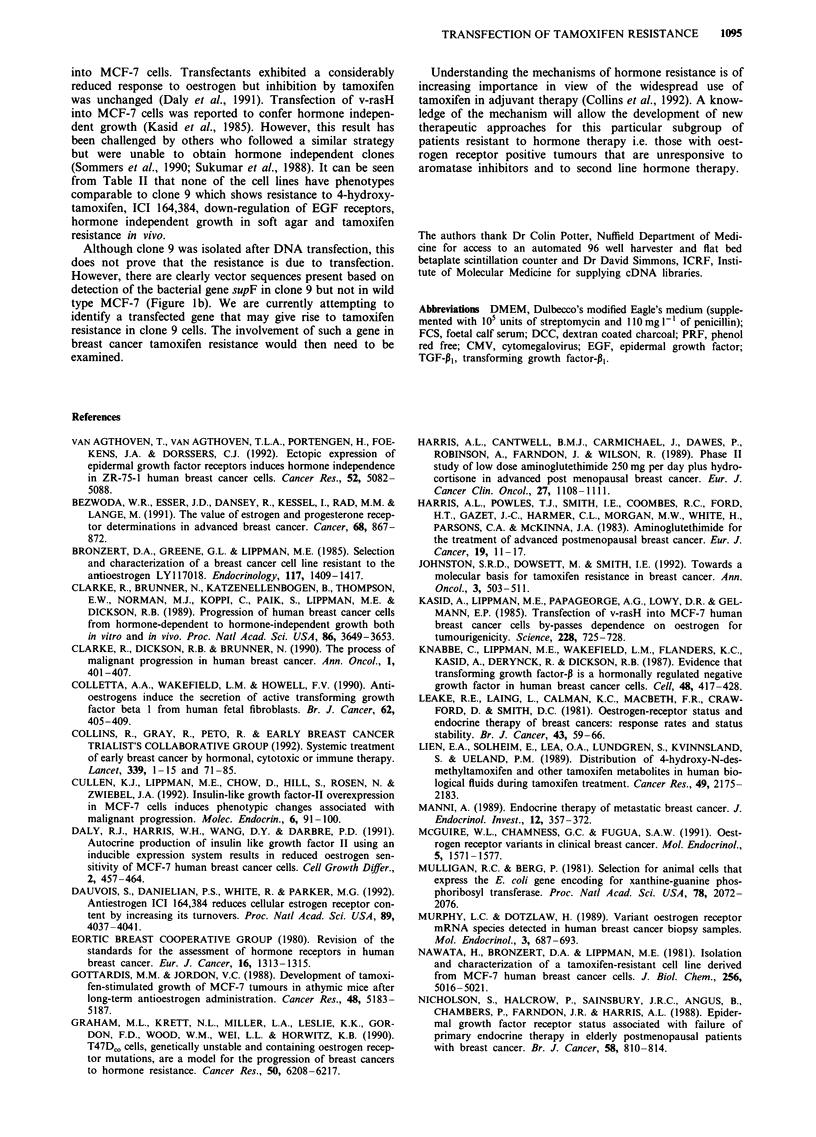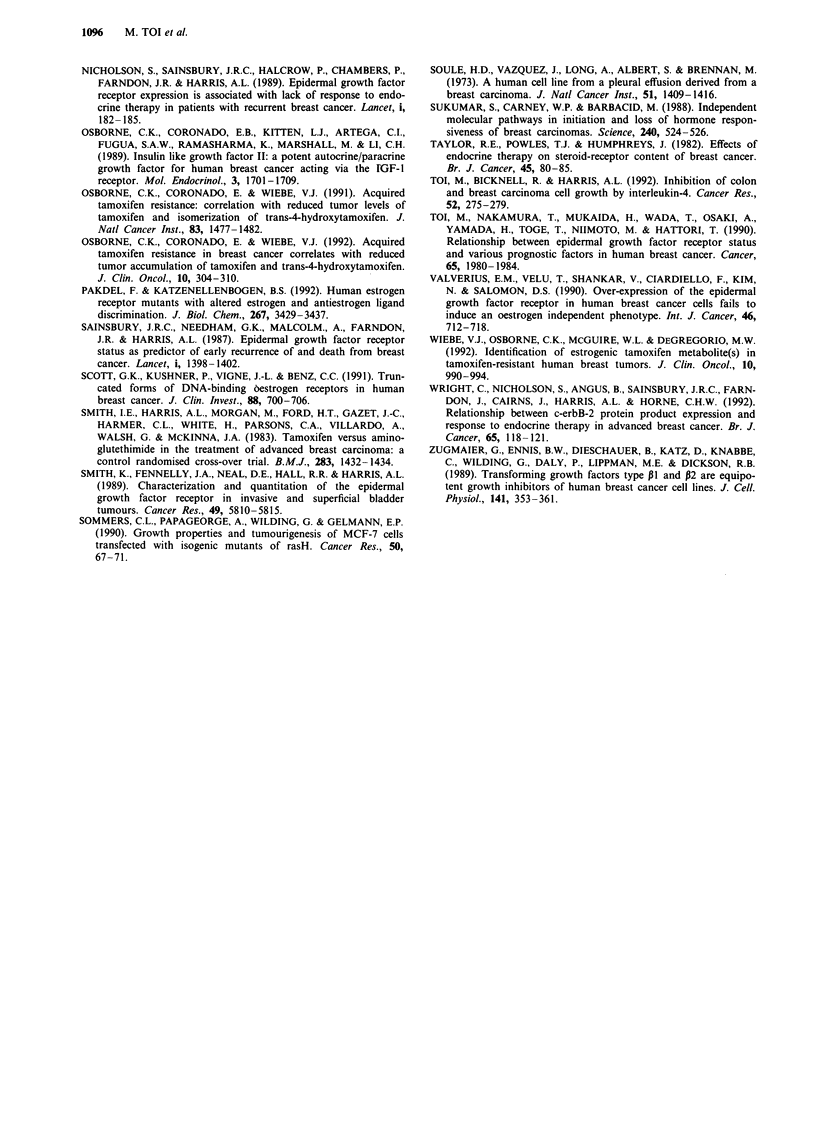# cDNA transfection followed by the isolation of a MCF-7 breast cell line resistant to tamoxifen in vitro and in vivo.

**DOI:** 10.1038/bjc.1993.486

**Published:** 1993-12

**Authors:** M. Toi, A. L. Harris, R. Bicknell

**Affiliations:** Molecular Oncology Laboratory, Imperial Cancer Research Fund, University of Oxford, John Radcliffe Hospital, UK.

## Abstract

**Images:**


 © Macmillan Press Ltd., 1993

### cDNA transfection followed by the isolation of a MCF-7 breast cell line resistant to tamoxifen *in vitro* and *in vivo*

M. Toi[1], A.L. Harris & R. Bicknell

*Molecular Oncology Laboratory, Imperial Cancer Research Fund, Institute of Molecular Medicine, University of Oxford, John Radcliffe Hospital, Oxford OX3 9DU, UK.*

**Summary** A tamoxifen resistant cell line (clone 9) has been isolated from the tamoxifen sensitive, hormone responsive MCF-7 breast carcinoma cell line after transfection with mixed cDNA libraries, followed by tamoxifen selection in the presence of oestrogens. Transfection was confirmed by Southern analysis with vector probes. Clone 9 in several-fold more resistant to tamoxifen and other anti-oestrogens than wild type cells when cultured either as a monolayer or as colonies in soft agar but retains oestrogen receptors. Clone 9 was less responsive to 17-β-oestradiol than were wild type MCF-7. In addition to showing *in vitro* tamoxifen resistance, clone 9 was also tamoxifen resistant *in vivo* when xenografted into the nude mouse. Culture medium conditioned by clone 9 cells stimulated quiescent cells of the same clone as well as wild type cells, whereas medium conditioned by wild type MCF-7 was inhibitory to both, suggesting that clone 9 may be secreting an autocrine growth factor. Clone 9 provides a novel model for further investigation of the mechanism of anti-oestrogen resistance that occurs without loss of oestrogen receptors. Preliminary results suggest that an autocrine growth stimulatory mechanism may be one pathway of such resistance.

Anti-hormonal therapy has proven a successful strategy in the treatment of breast cancer and its use in early disease prolongs survival. Thus, following surgery the anti-oestrogen tamoxifen is effective in extending both disease free and overall survival of primary breast cancer patients. Tamoxifen also induces tumour suppression in about 50% of the patients with advanced oestrogen receptor positive breast cancer (Collins *et al.*, 1992; Manni, 1989). A major drawback in the successful use of anti-oestrogen therapy has been the development of resistance to the drug. Nearly all patients with recurrent disease develop resistance after an initial response and in the case of adjuvant therapy, 40-50% of node positive cases still relapse and die. Some tamoxifen resistant tumours respond to a second line hormone manipulation (Harris *et al.*, 1983, 1989). In this case approximately half of the previous responders respond a second time (Smith *et al.*, 1983). In contrast, a second resistant subtype is unresponsive to all hormone manipulations despite continued expression of oestrogen receptors. The mechanisms of the latter resistance may include over expression of growth factors (Johnston *et al.*, 1992; Cullen *et al.*, 1992) or growth factor receptors (Nicholson *et al.*, 1988, 1989; Wright *et al.*, 1992), change in tamoxifen metabolism (Osborne *et al.*, 1992), mutations in the oestrogen receptor (Graham *et al.*, 1990; Pakdel & Katzenellenbogen, 1992) or oestrogen receptor splice variants (McGuire *et al.*, 1991; Murphy & Dotzlaw, 1989; Scott *et al.*, 1991).

To further examine resistance to tamoxifen in the presence of oestrogen receptors, we have attempted transfection of tamoxifen resistance into the tamoxifen sensitive MCF-7 cell line. In view of the possibility that novel autocrine growth pathways may lead to tamoxifen resistance, a mixture of several cDNA libraries from hormone unresponsive cells was used in the transfection. As stimulation of some cell types with cytokines is known to induce expression of cell adhesion molecules and secreted growth factors, cDNA libraries from such stimulated cells were used. Tamoxifen selection was carried out in the presence of oestrogens to approximate as closely as possible to the situation in which resistance arises *in vivo*. Tamoxifen levels similar to those achieved in plasma *in vivo* with conventional dosage regimens were used (Lien *et al.*, 1989). We now report isolation of tamoxifen resistant MCF-7 cells following cDNA transfection of such cDNA libraries. One of the clones (clone 9) retains oestrogen receptors and shows cross resistance to 4-hydroxytamoxifen as well as to the new pure anti-oestrogen ICI 164,384. Clone 9 is the first breast carcinoma line to show resistance to tamoxifen both *in vitro* and *in vivo* while continuing to express oestrogen receptors, and provides an *in vitro* model with which to investigate the mechanism of such resistance.

### Materials and methods

### *Materials*

$\alpha^{32}$P-dCTP and $^{3}$H-methylthymidine were from Amersham plc, Amersham, UK. TGF-$\beta_1$ was from British Biotechnology Ltd., Oxford, UK. 17-β-Oestradiol slow release pellets were from Innovative Research of America, Toledo, Ohio, USA. Routine laboratory chemicals were from Sigma or B.D.H. All tissue culture media were from the ICRF Central Services. Foetal calf serum was from J. Bio, Les Ulis, France. BALBc NuNu mice were bred and housed at the ICRF Clare Hall Laboratories, South Mimms, UK.

### Methods

### *Transfection of MCF-7 cells with cDNA libraries*

MCF-7 cells ($2 \times 10^7$) were detached by treatment with trypsin, pelleted and resuspended in 1 ml of DMEM containing 20 µg of the following cDNA libraries in pCDM8: U937 lymphoma; endotoxin, interleukin-1 and tumour necrosis factor-α activated human umbilical vein endothelial cells and proliferating (fibroblast growth factor stimulated) bovine adrenal capillary endothelial cells. For each library, one half (10 µg) of the DNA was linearised by overnight incubation with *Sfi*I prior to transfection. Co-transfection with 10 µg of pSV2-gpt containing the *E. coli* xanthine-guanine phosphoribosyl transferase gene (XGPRT) (*gpt* resistance gene) was carried out by electroporation with a capacitance of 25 µF as follows (i) control electroporation at 300 V (no exogenous DNA), (ii) *gpt* resistance plasmid alone at 300 V and (iii) cocktails with *gpt* resistance plasmid and the above libraries at the following voltages 300, 330, 360 and 390. After electroporation cells were seeded into a 15 cm petri dish in DMEM/10% FCS and left for 24 h to recover.

Correspondence: R. Bicknell.
[1]Present address: Department of Surgery, Tokyo Metropolitan Komagome Hospital, 3-18-22, Honkomagome, Bunkyo-ku, Tokyo, Japan.
Received 3 February 1993; and in revised form 22 July 1993.

*Sequential selection of transfectants for gpt and tamoxifen resistance. gpt selection*

After 24 h in DMEM/10% FCS, cells were subjected to mycophenolic acid selection in the following medium: DMEM/10% dialysed FCS supplemented with xanthine (250 $\mu$g ml$^{-1}$), hypoxanthine (15 $\mu$g ml$^{-1}$), thymidine (10 $\mu$g ml$^{-1}$), mycophenolic acid (15 $\mu$g ml$^{-1}$) and aminopterin (2 $\mu$g ml$^{-1}$) for 14 days (Mulligan & Berg, 1981). Then with the same media lacking aminopterin and with the mycophenolic acid concentration reduced to 10 $\mu$g ml$^{-1}$ for a further 14 days. At this point selection medium was removed and cells grown on for a further 10 days in DMEM/10% FCS by which time distinct colonies of transfectants were clearly visible. Typically each transfection gave rise to ~$10^4$ colonies (i.e. a transfection efficiency of 0.05%). All cells were dead in the control (no DNA) transfection. Transfection with 300 V was optimal.

*Tamoxifen selection*

On completion of mycophenolic acid selection, the clones from transfected libraries were subjected to tamoxifen selection *in situ* as follows: DMEM/10% FCS supplemented with tamoxifen (1 $\mu$M) for 14 days. Culture medium was replaced every 2 days. At this point the control *gpt* transfection contained a few small colonies. By contrast the 300 V library transfection contained ten rapidly growing colonies (i.e. tamoxifen resistance to one in two million) and the 330–390 V transfections a further ten colonies between them. All 20 colonies were removed by exposure to trypsin within stainless steel cloning rings and cultured in the continued presence of tamoxifen (1 $\mu$M) before freezing back. Clones 1, 3 and 9 showed the fastest growth in the presence of 1 $\mu$M tamoxifen and were selected for further study. After a further 3 months culture in the presence of tamoxifen (1 $\mu$M) they were phenotypically characterised.

*Characterisation of growth properties of transfectant clones relative to wild type MCF-7. $^3$H-methylthymidine uptake assay*

Mitogenesis assays were performed in 96 well tissue culture plates. MCF-7 wild type or transfectants were grown in oestrogen free conditions for 2 weeks before being seeded at 3,000 cells/well in 10% DCC treated FCS, left 24 h and then treated with 17-$\beta$-oestradiol (500 pM) with or without anti-oestrogen in fresh PRF DMEM/10% DCC treated FCS. 17-$\beta$-oestradiol (500 pM) and anti-oestrogens were present for a further 4 days at which time cells were pulse labelled with 0.5 $\mu$Ci of $^3$H-methylthymidine per well for 4 h before being removed by treatment with trypsin and harvested with an automated 96 well harvester directly onto filter mats and counted in a Pharmacia-Wallac flat bed betaplate scintillation counter. All experiments were repeated at least once with similar results.

*Cell counting to determine growth curves*

The indicated number of cells were seeded into six well tissue culture plates in PRF DMEM/10% DCC treated FCS. At appropriate times, cells were detached by exposure to trypsin and counted in a Coulter counter. Cells were fed with fresh PRF DMEM/10% DCC treated FCS, with or without inhibitors on days 3, 5 and 7.

*Colony formation in soft agar*

Three thousand cells were suspended in 300 $\mu$l of 0.3% 'Difco' noble agar in PRF DMEM/10% DCC treated FCS and layered over 0.3 ml of a 0.6% agar-medium basal layer in 24 well tissue culture plates. Samples were then fed with PRF DMEM/10% DCC treated FCS supplemented with tamoxifen (0, 1, 5 or 10 $\mu$M) and incubated at 37°C for 17 days. Half of the samples were also supplemented with 17-$\beta$-oestradiol (1 nM). On day 17 the number of colonies >30–40 $\mu$m in diameter was then counted by eye. The relative size of the colonies was also noted. All points were triplicates. Experiments were repeated twice.

*DNA extraction and Southern blotting*

MCF-7 or transfectants ($2 \times 10^7$ cells) were lifted with trypsin and pelleted. The pellet was washed with PBS ($\times$ 2) and then lysed with 4 ml of the following buffer 0.1 M NaCl, 0.01 M Tris-HCl, 0.025 M EDTA, pH 8 and 0.5% SDS. Proteinase K was added to 100 $\mu$g ml$^{-1}$ and the solution incubated at 50°C overnight. RNase A (1 $\mu$g ml$^{-1}$) was added, followed by a further incubation at 37°C for 1 h. The DNA was then extracted sequentially with phenol, phenol/chloroform (1:1) and then chloroform before dialysis against three changes of 10 mM Tris-HCl, 1 mM EDTA, pH 8. Isolated DNA was stored at 4°C.

Samples of DNA were restricted with *Spe*I/*Hind*III (to probe for the CMV promoter) with *Sac*II/*Spe*I (to probe for *sup*F) and with *Hind*III/*Bgl*II (to probe for *gpt*). DNA (10 $\mu$g per lane) was electrophoresed in 0.7% agarose gels and transferred to 'HyBond' nylon membranes by capillary blotting using 0.4 M NaOH as convectant. Filters were prehybridised overnight at 72°C in 10% dextran sulphate, 10 $\times$ Denhardts solution (270 mM NaCl, 15 mM sodium phosphate, 1.5 mM EDTA, pH 7), 2% SDS and salmon sperm DNA (400 $\mu$g ml$^{-1}$). Hybridisation was carried out overnight at 72°C under the same conditions as for pre-hybridisation, but including cDNA probes labelled by random priming. Filters were washed to a stringency of 0.1 $\times$ SSC, 1% SDS at 55°C and then exposed to preflashed film at −70°C. Probes were removed from filters by treatment with 0.4 M NaOH for 30 min at 45°C, followed by treatment with 0.2 M Tris-HCl, 0.1 $\times$ SSC, 0.1% SDS, pH 8 at 45°C for 30 min.

*Oestrogen and EGF receptor assay*

The oestrogen receptor number in oestrogen-depleted cells was determined by the DCC ligand binding assay in accord with EORTC guidelines (EORTC, 1980). Cytosolic fractions were prepared as previously described (Leake *et al.*, 1981) from subconfluent cells. EGF receptors were measured by a competitive binding assay on membrane fractions (Smith *et al.*, 1989).

*Conditioning of culture media by wild type MCF-7 and clone 9 transfectant*

Subconfluent cells were washed ($\times$ 3) with DMEM and then left for 48 h to condition DMEM. Media was collected and dialysed against water ($\times$ 3) with tubing of molecular weight cut-off 3.5 kDa. Protein was freeze dried, reconstituted with $\times$ 10 PRF DMEM and water to give a solution in PRF DMEM. $^3$H-methylthymidine assays were then performed as described above in 2% DCC treated FCS.

*Xenografts in BALBc NuNu mice*

BALBc NuNu mice were ovariectomised and received two subcutaneous implants. One a 60-day release pellet containing 1.5 mg 17-$\beta$-oestradiol and the other $10^7$ MCF-7 cells. The pellet was placed at the back of the neck and the cells in the flank. Half of the mice then received twice weekly s.c. injection of 100 $\mu$g tamoxifen in 200 $\mu$l of sesame oil. The controls received sesame oil alone. Tumour measurements were performed twice weekly.

### Results

*Transfection of MCF-7 cells and Southern analysis*

In preliminary experiments a tamoxifen selection protocol was developed such that of $2 \times 10^7$ wild type cells placed under selection, none survived and we were unable to determine the frequency of spontaneous resistance. MCF-7 can give rise to spontaneously anti-oestrogen resistant lines (see e.g. Nawata *et al.*, 1981) but it appears that many more cells have to be placed under selection. The selection protocol used here involved replacing the culture media every other day with fresh 10% FCS/DMEM containing 1 μM tamoxifen. After transfection of the cDNA libraries and mycophenolic acid selection the gpt control and the gpt/library transfection each gave rise to around $10^4$ discrete colonies. These were subjected directly to tamoxifen selection *in situ*. At 14 days the gpt control showed only two remaining colonies that did not appear to be proliferating. By contrast, the library transfectants containing 20 rapidly growing colonies. Thus, 20 resistant clones were obtained from $10^4$ transfectants, whereas wild type MCF-7 failed to give rise to any resistant clones from $2 \times 10^7$ cells. This argues strongly for resistance to have arisen as a result of the transfection event. Of the tamoxifen resistant transfectants, clones 1, 3 and in particular 9 were fast growing in the presence of 1 μM tamoxifen. These three clones were chosen for further characterisation. Figure 1 shows Southern analysis of DNA from these cells probed with (a) CMV promoter and (b) *sup*F. Probing for the CMV promoter (Figure 1a) gave a strong positive in clone 9, however, a weak band was also present in wild type cells, suggesting that they may contain CMV promoter sequences. The lane labelled T corresponds to a digest of DNA from a transient transfection of HL60 cells with pCDM8 and P to a digest of the plasmid pCDM8. In view of the weak signal for CMV sequences in wild type MCF-7, we also probed for the bacterial gene *sup*F. Figure 1b shows that *sup*F is clearly present in clones 3 and 9 but absent from wild type MCF-7. We conclude that clones 3 and 9 are stable transfectants. Probing for *gpt* showed this to be absent in wild type MCF-7, but present in all transfectants (data not shown), consistent with their growth under mycophenolic acid selection.

### *Effect of the anti-oestrogens tamoxifen, 4-hydroxytamoxifen and ICI 164,384 on $^3$H-methylthymidine uptake and growth of MCF-7 wild type and clone 9 cells*

The effect of 17-β-oestradiol and of anti-oestrogens on $^3$H-methylthymidine uptake and on growth of MCF-7 wild type and clone 9 cells was examined in PRF DMEM/10% DCC treated FCS after culture of the cells in this media for 2 weeks to deprive them of oestrogens. Figure 2 shows dose response curves for the inhibition of 17-β-oestradiol (500 pM) stimulated $^3$H-methylthymidine uptake by (a) tamoxifen, (b) 4-hydroxytamoxifen and (c) ICI 164,384. $^3$H-methylthymidine uptake was measured by pulse labelling at the end of the experiment to avoid any effects of variation in growth rates in the first few days of the experiment. Clone 9 was seen to be markedly more resistant to the growth inhibitory effect of tamoxifen and 4-hydroxytamoxifen than were wild type cells. Substantial inhibition of wild type growth was seen in $10^{-8}$ M 4-hydroxytamoxifen whereas higher concentrations of 4-hydroxytamoxifen ($10^{-6}$ M) were required to see similar inhibition in clone 9. Clone 9 is more resistant to ICI 164,384 than wild type cells but appears less so than it is to the other two anti-oestrogens. Growth curves confirmed that the effects on $^3$H-methylthymidine uptake reflect true effects on cell growth. Thus, Figure 3a shows that wild type cells are stimulated by 17-β-oestradiol and that this stimulation is blocked by tamoxifen (1 μM), 4-hydroxytamoxifen (100 nM) or ICI 164,384 (100 nM). The observation that tamoxifen

Figure 1 Southern analysis of transfectants. **a**, Probing for the CMV promoter. Left to right: wild-type MCF-7, clone 9, clone 3, clone 1, DNA from a pCDM8 transient transfection into HL60 cells (T) and a digest of plasmid CDM8 (P). **b**, Probing for the *sup*F gene. Left to right: clone 9, clone 3, digest of pCDM8, wild type MCF-7, transient transfection of HL60 cells with pCDM8 containing the gene for CD31 (T) and finally a digest of plasmid CDM8 (P).

Figure 2 Anti-oestrogen inhibition of 17-β-oestradiol (500 pM) stimulated $^3$H-methylthymidine uptake by MCF-7 wild type (O) and MCF-7 clone 9 (●) transfectant. **a**, tamoxifen, **b**, 4-hydroxytamoxifen and **c**, ICI 164,384. For conditions see methods. (ordinate: mean c.p.m. per well ± s.d., $n = 10$ where error bars are not shown they fell within the size of the symbol.)

**Figure 3** Effect of anti-oestrogens on growth curves for MCF-7 wild type and MCF-7 clone 9 transfectant. (○) control growth (i.e. no added 17-β-oestradiol), (▲) with 17-β-oestradiol (500 pM), (□) with 17-β-oestradiol and tamoxifen (1 μM), (■) with 17-β-oestradiol and 4-hydroxytamoxifen (100 nM) and (Δ) with 17-β-oestradiol and ICI 164,384 (100 nM). Cells were oestrogen depleted by culture in PRF DMEM/10% DCC treated FCS for 2 weeks before seeding at day 1. Cells were re-fed with fresh hormone and/or anti-oestrogen in PRF DMEM/10% DCC treated FCS on days 3, 5 and 7 (mean ± s.d., $n = 2$).

**Figure 4** Dose response of $^3$H-methylthymidine uptake by MCF-7 wild type (○) and MCF-7 clone 9 transfectant (●) in response to 17-β-oestradiol. Two week oestrogen depleted cells were seeded at 3,000 cells/well and treated with 17-β-oestradiol for 4 days in PRF DMEM/5% DCC treated FCS. Cells were pulse labelled for 4 h with $^3$H-methylthymidine before harvesting (mean, ± s.d., $n = 10$).

reduces proliferation below that in the PRF DMEM control suggests that there remains residual oestrogen in the culture medium. Figure 3b shows that oestrogen had no detectable effect on the rate of growth of clone 9. By contrast, clone 9 continues to show substantial growth in the presence of all three anti-oestrogens. The growth in the presence of ICI 164,384 is particularly interesting in view of the lower level of resistance shown by clone 9 in the $^3$H-methylthymidine uptake assay (Figure 2c). To confirm that clone 9 is less responsive to oestrogen we examined $^3$H-methylthymidine uptake in response to oestradiol. Figure 4 shows that the percentage stimulation of clone 9 by oestradiol is less (+8%) than that maximally seen with MCF-7 wild type cells (+25%). This accords with the poor stimulation of clone 9 growth by 17-β-oestradiol (Figure 3b).

*Colony formation in soft agar*

The growth of MCF-7 wild type and clone 9 cells in soft agar was examined in PRF DMEM/10% DCC treated FCS in the presence of 0, 1 and 5 μM tamoxifen (Figure 5a). Half of the samples also received 17-β-oestradiol (1 nM). Differences were apparent between the wild type and clone 9 cells. Not only were there many more colonies of clone 9 cells but the colony size was larger relative to controls (Figure 6). Clone 9 also formed colonies in the presence of 5 μM tamoxifen (Figure 6c), a concentration of tamoxifen at which wild type cells showed no growth (data not shown). Colony formation by MCF-7 wild type cells was stimulated by 17-β-oestradiol; but not that of clone 9 which grew equally fast in the absence of oestrogens (Figure 5a). However, the growth of clone 9 became oestrogen responsive when antagonised by the presence of either 1 or 5 μM tamoxifen (Figure 5a).

These findings raise the possibility that the resistance of clone 9 to 1 and 5 μM tamoxifen is due simply to its faster growth rate or greater colony forming ability. This is not the case, for, as shown in Figure 5b, after normalisation of the number of colonies, clone 9 is still markedly less inhibited by 1 and 5 μM tamoxifen. Further experiments examined colony formation in the presence of 4-hydroxytamoxifen and ICI 164,384 both on supplementation with 17-β-oestradiol (1 nM) or in PRF DMEM/DCC treated FCS. In each case, clone 9 was substantially more resistant than either wild type or clone 3 (data not shown). Resistance was particularly striking in the absence of 17-β-oestradiol. Colony formation by clone 9 in the absence of oestragens may be a component of the tamoxifen resistant phenotype.

*Effect of media conditioned by wild type MCF-7 and clone 9 on $^3$H-methylthymidine uptake by both cell lines*

DMEM was conditioned by cultures of MCF-7 wild type and clone 9 cells under identical conditions. These media were then dialysed, freeze dried and reconstituted to give a solution in PRF DMEM/2% DCC treated FCS. These media were tested for their effects on $^3$H-methylthymidine uptake in both MCF-7 wild type and clone 9 cells. Figure 7 shows that medium conditioned by wild type MCF-7 was inhibitory to both wild type MCF-7 and clone 9. This presumably reflects the activity of inhibitory cytokines outweighing that of stimulatory cytokines e.g. MCF-7 are known to produce and to be inhibited by TGF-$\beta_1$ (Zugmaier *et al.*, 1989; Knabbe *et al.*, 1987). In contrast medium conditioned by clone 9 stimulated $^3$H-methylthymidine uptake in both cell types when compared to PRF DMEM/2% DCC treated FCS controls. We conclude that transfection has induced secretion of a mitogen (or reduced secretion of an inhibitor) by the MCF-7 cells.

*Oestrogen and EGF receptor expression*

The data in Table I shows that there was a small decrease in the oestrogen and a somewhat larger decrease in the EGF

**Figure 5** Growth of MCF-7 wild type and transfectant clones 1, 3 and 9 in soft agar. Assays were carried out as described in Methods either with or without 1 nM 17-β-oestradiol supplementation during the growth of the colonies. **a**, colony number; **b**, Inhibition by anti-oestrogens (in the presence of 1 nM 17-β-oestradiol) normalised to the number of colonies formed by MCF-7 wild type cells. Wild type (○) and clone 9 (●).

receptors in those transfectants examined (including clone 9) when compared to wild type cells. However, in each case both receptors were clearly detectable.

### *Xenografts in BALBc NuNu mice*

Neither wild type MCF-7 nor clone 9 cells form tumours when injected s.c. ($10^7$ cells) into ovariectomised BALBc NuNu mice that had not received a slow release oestrogen implant. In contrast, when the mice also received an oestrogen implant, slow growing tumours formed. If the mice also received tamoxifen, tumours from both wild type and clone 9 between days 15 and 25 grew significantly faster than those in control mice (Figure 8). However, beyond day 30 of implantation marked differences became apparent. Tumours from wild type cells ceased growing and subsequently started to regress. By day 53 two tumours had become immeasurably small and the analysis was terminated. In contrast, tumours from clone 9 cells continued to grow up to day 47 (end of experiment) at a rate that was indistinguishable from clone 9 tumours in mice that did not receive tamoxifen. We note that the tumours formed by clone 9 cells were all significantly smaller than those formed by wild type MCF-7. The reason for this is not known.

### Discussion

The tamoxifen resistant clone 9, isolated after cDNA transfection, shows an *in vitro* phenotype similar to one of the major types of clinical anti-oestrogen resistance, namely, tamoxifen resistance in the presence of expressed oestrogen receptors. Patients with recurrent disease and oestrogen receptor positive tumours respond well to tamoxifen (approx-

**Figure 6** Growth of MCF-7 wild type **a**, and clone 9 transfectant **b**, in soft agar in the presence of 1 μM tamoxifen. Growth of clone 9 transfectant in the presence of 5 μM tamoxifen **c**. In each case 1 nM 17-β-oestradiol was added to PRF DMEM/10% DCC treated FCS.

**Figure 7** $^{3}$H-Methylthymidine uptake in response to media conditioned by MCF-7 wild type cells and clone 9 by cells of the same lineage. Dialysed and freeze dried conditioned medium was reconstituted in PRF DMEM/2% DCC treated FCS. Cells were exposed to media for 48 h before harvesting (mean, ±s.e.m., $n = 10$). In each case the increase over 2% FBS seen with clone 9 conditioned media and decrease seen with wild type conditioned media was highly significant ($P < 0.001$). The experiment was repeated three times with similar results.

**Table I** Expression of oestrogen and EGF receptors in wild type and transfected MCF-7 cells

| *Cell line* | *Oestrogen receptor fmol mg$^{-1}$ protein* | *EGF receptor fmol mg$^{-1}$ protein* |
|---|---|---|
| Wild type | 79 | 5.8 |
| Clone 1 | 36 | 0.1 |
| Clone 3 | 52 | not done |
| Clone 9 | 30 | 1.4 |

Receptors were assayed by a competitive ligand binding assay. Cells were cultured in PRF DMEM/10% DCC treated FCS for 2 weeks prior to oestrogen receptor assay.

imately 50–60% response rate). While the majority relapse within 2 years, if treated with a second anti-hormonal therapy, approximately 50% may respond again (Smith *et al.*, 1983). Oestrogen receptors are detectable in patients relapsing on tamoxifen, but not all of these respond to second line therapy (Leake *et al.*, 1981; Taylor *et al.*, 1982). It is this latter group of patients with oestrogen receptor positive tumours failing tamoxifen after an initial response, and not responding to further endocrine manipulation, who present a major clinical problem. Clone 9 has a phenotype that makes it a suitable model with which to investigate the mechanism(s) of such resistance. The parent MCF-7 cells that were transfected are hormone responsive and anti-oestrogen sensitive. Clone 9 responds to anti-oestrogens but requires higher doses for inhibition than do wild type cells. Clone 9 also grows *in vitro* but not *in vivo* in the absence of oestrogen. The latter apparent discrepancy indicates that while oestrogen is not needed for growth of clone 9 it is needed for successful tumour formation. It is possible that oestrogen stimulates release of, for example, an angiogenic factor or protease (e.g. stromolysin) which is essential for tumour formation but that has no effect on the *in vitro* growth rate of the cells.

Oestrogen receptors were detectable in clone 9 by ligand binding (Table I) and we conclude that a major deficiency in hormone binding does not account for resistance. Although oestrogen receptors were lower in clone 9, the level of expression is well within the range associated with hormone responsiveness (Bezwoda *et al.*, 1991). Further, resistance to three anti-oestrogens, including ICI 164,384 which is thought to exert its anti-oestrogenic effect by a different mechanism to that of tamoxifen (Dauvois *et al.*, 1992), is consistent with resistance not arising from mutation in the oestrogen receptor.

Inhibitory growth factors are an important pathway by which tamoxifen is thought to mediate its effect (Knabbe *et al.*, 1987; Colletta *et al.*, 1990). The secretion of active TGF-$\beta_1$, an inhibitor of breast carcinoma cells in culture (Zugmaier *et al.*, 1989; Knabbe *et al.*, 1987; Toi *et al.*, 1992) is induced by tamoxifen (Knabbe *et al.*, 1987) and loss of TGF-$\beta_1$ receptors could produce tamoxifen resistance. However, both wild type MCF-7 and clone 9 were similarly inhibited by TGF-$\beta_1$ (data not shown) and this fails to explain the resistance that we have observed here.

It has been shown that the expression of several growth factors and growth factor receptors is markedly changed in anti-oestrogen resistant tumours, for example, we have

**Figure 8** Growth of wild type MCF-7 and clone 9 transfectant in BALBc NuNu mice. Mice were ovariectomised and received a 60 day slow release oestrogen pellet implant. Tamoxifen treated mice received a twice weekly s.c. dose of 100 μg tamoxifen in 200 μl of sesame oil. Control (○), tamoxifen treated (●) (mean, ± s.e.m., wild type $n = 10$, clone 9, $n = 5$). The experiment was repeated twice with similar results.

previously shown high EGF receptor and c-*erb*B-2 expression in hormone resistant primary breast cancer (Nicholson *et al.*, 1988, 1989; Wright *et al.*, 1992; Sainsbury *et al.*, 1987; Toi *et al.*, 1990). There is also an inverse relationship between oestrogen and EGF receptor expression in breast cancer and a switch to an EGF receptor positive phenotype (up-regulation of EGF receptor) may be associated with hormone resistance. In clone 9, however, the EGF receptor level was lower than in the parental cells, as was the case in the other less tamoxifen resistant clones for which EGF receptor expression was determined.

The experiments in which medium was conditioned by wild type MCF-7 or clone 9 and then reconstituted in PRF DMEM/2% DCC treated FCS and administered back to quiescent cells suggest that an enhanced autocrine loop could account for the hormone independent growth. Medium conditioned by clone 9 cells stimulated wild-type and clone 9, whereas medium from MCF-7 wild-type cells was inhibitory to both types (Figure 7). Candidate growth factors could be TGF-β, insulin like growth factors, or fibroblast like growth factors all of which have been reported to stimulate growth of breast carcinoma cells.

The degree of resistance demonstrated by clone 9 varied depending on the endpoint selected, but in all assays was significantly more resistant than wild-type MCF-7. Thus, in the inhibition of $^3$H-methylthymidine uptake $IC_{50}$ differs by a factor of ten between the two cell lines. Inhibition at 1 μM tamoxifen, the plasma level found with conventional dosing (Lien *et al.*, 1989), was 20% for clone 9 *vs* 100% for wild type MCF-7 i.e. a 5-fold difference. When growth curves were determined by cell counting, inhibition at day 9 of oestrogen stimulated growth was complete with wild type cells but clone 9 was unaffected (Figure 3).

Oestrogen hypersensitivity could also account for some of the featues of clone 9, with minute amounts residual in DCC treated FCS or carried over in cells causing apparent hormone 'independent' growth. Dose response curves to oestradiol showed this was not the case, however, and that clone 9 actually has a reduced response to 17-β-oestradiol (Figure 4), a result in accord with the growth curve shown in Figure 3b. Tamoxifen stimulation is another reported mechanism of tamoxifen resistance *in vivo*, but *in vitro* the growth curves clearly show no such effect in clone 9. It is possible that downstream pathways normally regulated by oestrogen have become constitutively activated and it would be of interest to analyse oestrogen regulated genes such as pS2, cathepsin D, and the oestrogen receptor in this transfectant. The results with the pure anti-oestrogen suggest that some patients relapsing after tamoxifen therapy may not benefit from a second hormonal therapy. Alterations in tamoxifen metabolism or poor tamoxifen uptake are other mechanisms of resistance (Osborne *et al.*, 1991, 1992; Weibe *et al.*, 1992). However, they cannot account for hormone independent growth in agar, and the fact that the clone 9 cell line is resistant to an entirely different structural class of anti-oestrogen.

Previously, several other tamoxifen resistant or hormone independent cell lines have been isolated by exposure to selecting drugs or by transfection of oncogenes. Their phenotypes are summarised in Table II. Clinical studies have correlated high level EGF receptor expression with hormone independent growth of human breast cancer (Nicholson *et al.*, 1988, 1989). However, clone 9 MCF-7 cells show hormone independent growth but a decrease in expression of EGF receptors relative to the hormone dependent wild-type cells. Similarly spontaneously tamoxifen resistant lines such as R-27 and LY2 have EGF receptor numbers per cell that fall well within the range of wild type MCF-7. To examine whether overexpression of the EGF receptor leads to hormone independent growth, the EGF receptor was transfected into the hormone dependent human breast carcinoma line ZR75. The results are controversial. Thus, while one study (Valverius *et al.*, 1990) claims that high level expression of the receptor failed to alter hormone dependency or tamoxifen sensitivity, a second study (van Agthoven *et al.*, 1992) has disagreed. We conclude that *in vivo* EGF receptor expression plays a complex and possibly paracrine role in the evolution of hormone independent growth.

IGF-II which is known to be a growth factor for breast carcinoma cells (Osborne *et al.*, 1989) has been transfected

**Table II** Tamoxifen resistant and/or hormone independent cell lines isolated from MCF-7 cells

| | *In vitro* | | *Soft agar* | | *Xenograft* | |
|---|---|---|---|---|---|---|
| *Cell line* | H.D. | T.S. | H.D. | T.S. | H.D. | T.S. |
| MCF-7 WT[a] | + | + | + | + | + | + |
| *Group A* | | | | | | |
| R-27[b] | – | – | N.D. | N.D. | + | + |
| BSK-2 and -3[c] | – | + | N.D. | N.D. | – | N.D. |
| LY-2[d] | + | – | N.D. | N.D. | Not tumourigenic | |
| *Group B* | | | | | | |
| M-III[e] | – | + | – | + | – | + |
| MCF-TAM[f] | + | + | N.D. | N.D. | E2 and TAM dependent | |
| MCF-7 ADR[g] | – | – | N.D. | N.D. | – | N.D. |
| *Group C* | | | | | | |
| Clone 9 | – | – | – | – | + | – |

Abbreviations: H.D., hormone dependent; T.S. tamoxifen sensitive; N.D., not determined. [a]Soule *et al.* (1973); [b]Nawata *et al.* (1981); [c]Clarke *et al.* (1989); [d]Bronzert *et al.* (1985); [e]Clarke *et al.* (1990); [f]Gottardis *et al.* (1988); [g]M. Toi, A.L. Harris & R. Bicknell – unpublished data.

into MCF-7 cells. Transfectants exhibited a considerably reduced response to oestrogen but inhibition by tamoxifen was unchanged (Daly *et al.*, 1991). Transfection of v-rasH into MCF-7 cells was reported to confer hormone independent growth (Kasid *et al.*, 1985). However, this result has been challenged by others who followed a similar strategy but were unable to obtain hormone independent clones (Sommers *et al.*, 1990; Sukumar *et al.*, 1988). It can be seen from Table II that none of the cell lines have phenotypes comparable to clone 9 which shows resistance to 4-hydroxytamoxifen, ICI 164,384, down-regulation of EGF receptors, hormone independent growth in soft agar and tamoxifen resistance *in vivo*.

Although clone 9 was isolated after DNA transfection, this does not prove that the resistance is due to transfection. However, there are clearly vector sequences present based on detection of the bacterial gene *sup*F in clone 9 but not in wild type MCF-7 (Figure 1b). We are currently attempting to identify a transfected gene that may give rise to tamoxifen resistance in clone 9 cells. The involvement of such a gene in breast cancer tamoxifen resistance would then need to be examined.

Understanding the mechanisms of hormone resistance is of increasing importance in view of the widespread use of tamoxifen in adjuvant therapy (Collins *et al.*, 1992). A knowledge of the mechanism will allow the development of new therapeutic approaches for this particular subgroup of patients resistant to hormone therapy i.e. those with oestrogen receptor positive tumours that are unresponsive to aromatase inhibitors and to second line hormone therapy.

The authors thank Dr Colin Potter, Nuffield Department of Medicine for access to an automated 96 well harvester and flat bed betaplate scintillation counter and Dr David Simmons, ICRF, Institute of Molecular Medicine for supplying cDNA libraries.

**Abbreviations** DMEM, Dulbecco's modified Eagle's medium (supplemented with $10^5$ units of streptomycin and $110 \text{ mg l}^{-1}$ of penicillin); FCS, foetal calf serum; DCC, dextran coated charcoal; PRF, phenol red free; CMV, cytomegalovirus; EGF, epidermal growth factor; TGF-$\beta_1$, transforming growth factor-$\beta_1$.

### References

VAN AGTHOVEN, T., VAN AGTHOVEN, T.L.A., PORTENGEN, H., FOEKENS, J.A. & DORSSERS, C.J. (1992). Ectopic expression of epidermal growth factor receptors induces hormone independence in ZR-75-1 human breast cancer cells. *Cancer Res.*, **52**, 5082–5088.

BEZWODA, W.R., ESSER, J.D., DANSEY, R., KESSEL, I., RAD, M.M. & LANGE, M. (1991). The value of estrogen and progesterone receptor determinations in advanced breast cancer. *Cancer*, **68**, 867–872.

BRONZERT, D.A., GREENE, G.L. & LIPPMAN, M.E. (1985). Selection and characterization of a breast cancer cell line resistant to the antioestrogen LY117018. *Endocrinology*, **117**, 1409–1417.

CLARKE, R., BRUNNER, N., KATZENELLENBOGEN, B., THOMPSON, E.W., NORMAN, M.J., KOPPI, C., PAIK, S., LIPPMAN, M.E. & DICKSON, R.B. (1989). Progression of human breast cancer cells from hormone-dependent to hormone-independent growth both *in vitro* and *in vivo*. *Proc. Natl Acad. Sci. USA*, **86**, 3649–3653.

CLARKE, R., DICKSON, R.B. & BRUNNER, N. (1990). The process of malignant progression in human breast cancer. *Ann. Oncol.*, **1**, 401–407.

COLLETTA, A.A., WAKEFIELD, L.M. & HOWELL, F.V. (1990). Antioestrogens induce the secretion of active transforming growth factor beta 1 from human fetal fibroblasts. *Br. J. Cancer*, **62**, 405–409.

COLLINS, R., GRAY, R., PETO, R. & EARLY BREAST CANCER TRIALIST'S COLLABORATIVE GROUP (1992). Systemic treatment of early breast cancer by hormonal, cytotoxic or immune therapy. *Lancet*, **339**, 1–15 and 71–85.

CULLEN, K.J., LIPPMAN, M.E., CHOW, D., HILL, S., ROSEN, N. & ZWIEBEL, J.A. (1992). Insulin-like growth factor-II overexpression in MCF-7 cells induces phenotypic changes associated with malignant progression. *Molec. Endocrin.*, **6**, 91–100.

DALY, R.J., HARRIS, W.H., WANG, D.Y. & DARBRE, P.D. (1991). Autocrine production of insulin like growth factor II using an inducible expression system results in reduced oestrogen sensitivity of MCF-7 human breast cancer cells. *Cell Growth Differ.*, **2**, 457–464.

DAUVOIS, S., DANIELIAN, P.S., WHITE, R. & PARKER, M.G. (1992). Antiestrogen ICI 164,384 reduces cellular estrogen receptor content by increasing its turnovers. *Proc. Natl Acad. Sci. USA*, **89**, 4037–4041.

EORTIC BREAST COOPERATIVE GROUP (1980). Revision of the standards for the assessment of hormone receptors in human breast cancer. *Eur. J. Cancer*, **16**, 1313–1315.

GOTTARDIS, M.M. & JORDON, V.C. (1988). Development of tamoxifen-stimulated growth of MCF-7 tumours in athymic mice after long-term antioestrogen administration. *Cancer Res.*, **48**, 5183–5187.

GRAHAM, M.L., KRETT, N.L., MILLER, L.A., LESLIE, K.K., GORDON, F.D., WOOD, W.M., WEI, L.L. & HORWITZ, K.B. (1990). T47D$_{co}$ cells, genetically unstable and containing oestrogen receptor mutations, are a model for the progression of breast cancers to hormone resistance. *Cancer Res.*, **50**, 6208–6217.

HARRIS, A.L., CANTWELL, B.M.J., CARMICHAEL, J., DAWES, P., ROBINSON, A., FARNDON, J. & WILSON, R. (1989). Phase II study of low dose aminoglutethimide 250 mg per day plus hydrocortisone in advanced post menopausal breast cancer. *Eur. J. Cancer Clin. Oncol.*, **27**, 1108–1111.

HARRIS, A.L., POWLES, T.J., SMITH, I.E., COOMBES, R.C., FORD, H.T., GAZET, J.-C., HARMER, C.L., MORGAN, M.W., WHITE, H., PARSONS, C.A. & MCKINNA, J.A. (1983). Aminoglutethimide for the treatment of advanced postmenopausal breast cancer. *Eur. J. Cancer*, **19**, 11–17.

JOHNSTON, S.R.D., DOWSETT, M. & SMITH, I.E. (1992). Towards a molecular basis for tamoxifen resistance in breast cancer. *Ann. Oncol.*, **3**, 503–511.

KASID, A., LIPPMAN, M.E., PAPAGEORGE, A.G., LOWY, D.R. & GELMANN, E.P. (1985). Transfection of v-rasH into MCF-7 human breast cancer cells by-passes dependence on oestrogen for tumourigenicity. *Science*, **228**, 725–728.

KNABBE, C., LIPPMAN, M.E., WAKEFIELD, L.M., FLANDERS, K.C., KASID, A., DERYNCK, R. & DICKSON, R.B. (1987). Evidence that transforming growth factor-$\beta$ is a hormonally regulated negative growth factor in human breast cancer cells. *Cell*, **48**, 417–428.

LEAKE, R.E., LAING, L., CALMAN, K.C., MACBETH, F.R., CRAWFORD, D. & SMITH, D.C. (1981). Oestrogen-receptor status and endocrine therapy of breast cancers: response rates and status stability. *Br. J. Cancer*, **43**, 59–66.

LIEN, E.A., SOLHEIM, E., LEA, O.A., LUNDGREN, S., KVINNSLAND, S. & UELAND, P.M. (1989). Distribution of 4-hydroxy-N-desmethyltamoxifen and other tamoxifen metabolites in human biological fluids during tamoxifen treatment. *Cancer Res.*, **49**, 2175–2183.

MANNI, A. (1989). Endocrine therapy of metastatic breast cancer. *J. Endocrinol. Invest.*, **12**, 357–372.

MCGUIRE, W.L., CHAMNESS, G.C. & FUGUA, S.A.W. (1991). Oestrogen receptor variants in clinical breast cancer. *Mol. Endocrinol.*, **5**, 1571–1577.

MULLIGAN, R.C. & BERG, P. (1981). Selection for animal cells that express the *E. coli* gene encoding for xanthine-guanine phosphoribosyl transferase. *Proc. Natl Acad. Sci. USA*, **78**, 2072–2076.

MURPHY, L.C. & DOTZLAW, H. (1989). Variant oestrogen receptor mRNA species detected in human breast cancer biopsy samples. *Mol. Endocrinol.*, **3**, 687–693.

NAWATA, H., BRONZERT, D.A. & LIPPMAN, M.E. (1981). Isolation and characterization of a tamoxifen-resistant cell line derived from MCF-7 human breast cancer cells. *J. Biol. Chem.*, **256**, 5016–5021.

NICHOLSON, S., HALCROW, P., SAINSBURY, J.R.C., ANGUS, B., CHAMBERS, P., FARNDON, J.R. & HARRIS, A.L. (1988). Epidermal growth factor receptor status associated with failure of primary endocrine therapy in elderly postmenopausal patients with breast cancer. *Br. J. Cancer*, **58**, 810–814.

NICHOLSON, S., SAINSBURY, J.R.C., HALCROW, P., CHAMBERS, P., FARNDON, J.R. & HARRIS, A.L. (1989). Epidermal growth factor receptor expression is associated with lack of response to endocrine therapy in patients with recurrent breast cancer. *Lancet*, **i**, 182–185.

OSBORNE, C.K., CORONADO, E.B., KITTEN, L.J., ARTEGA, C.I., FUGUA, S.A.W., RAMASHARMA, K., MARSHALL, M. & LI, C.H. (1989). Insulin like growth factor II: a potent autocrine/paracrine growth factor for human breast cancer acting via the IGF-1 receptor. *Mol. Endocrinol.*, **3**, 1701–1709.

OSBORNE, C.K., CORONADO, E. & WIEBE, V.J. (1991). Acquired tamoxifen resistance: correlation with reduced tumor levels of tamoxifen and isomerization of trans-4-hydroxytamoxifen. *J. Natl Cancer Inst.*, **83**, 1477–1482.

OSBORNE, C.K., CORONADO, E. & WIEBE, V.J. (1992). Acquired tamoxifen resistance in breast cancer correlates with reduced tumor accumulation of tamoxifen and trans-4-hydroxytamoxifen. *J. Clin. Oncol.*, **10**, 304–310.

PAKDEL, F. & KATZENELLENBOGEN, B.S. (1992). Human estrogen receptor mutants with altered estrogen and antiestrogen ligand discrimination. *J. Biol. Chem.*, **267**, 3429–3437.

SAINSBURY, J.R.C., NEEDHAM, G.K., MALCOLM., A., FARNDON, J.R. & HARRIS, A.L. (1987). Epidermal growth factor receptor status as predictor of early recurrence of and death from breast cancer. *Lancet*, **i**, 1398–1402.

SCOTT, G.K., KUSHNER, P., VIGNE, J.-L. & BENZ, C.C. (1991). Truncated forms of DNA-binding öestrogen receptors in human breast cancer. *J. Clin. Invest.*, **88**, 700–706.

SMITH, I.E., HARRIS, A.L., MORGAN, M., FORD, H.T., GAZET, J.-C., HARMER, C.L., WHITE, H., PARSONS, C.A., VILLARDO, A., WALSH, G. & MCKINNA, J.A. (1983). Tamoxifen versus aminoglutethimide in the treatment of advanced breast carcinoma: a control randomised cross-over trial. *B.M.J.*, **283**, 1432–1434.

SMITH, K., FENNELLY, J.A., NEAL, D.E., HALL, R.R. & HARRIS, A.L. (1989). Characterization and quantitation of the epidermal growth factor receptor in invasive and superficial bladder tumours. *Cancer Res.*, **49**, 5810–5815.

SOMMERS, C.L., PAPAGEORGE, A., WILDING, G. & GELMANN, E.P. (1990). Growth properties and tumourigenesis of MCF-7 cells transfected with isogenic mutants of rasH. *Cancer Res.*, **50**, 67–71.

SOULE, H.D., VAZQUEZ, J., LONG, A., ALBERT, S. & BRENNAN, M. (1973). A human cell line from a pleural effusion derived from a breast carcinoma. *J. Natl Cancer Inst.*, **51**, 1409–1416.

SUKUMAR, S., CARNEY, W.P. & BARBACID, M. (1988). Independent molecular pathways in initiation and loss of hormone responsiveness of breast carcinomas. *Science*, **240**, 524–526.

TAYLOR, R.E., POWLES, T.J. & HUMPHREYS, J. (1982). Effects of endocrine therapy on steroid-receptor content of breast cancer. *Br. J. Cancer*, **45**, 80–85.

TOI, M., BICKNELL, R. & HARRIS, A.L. (1992). Inhibition of colon and breast carcinoma cell growth by interleukin-4. *Cancer Res.*, **52**, 275–279.

TOI, M., NAKAMURA, T., MUKAIDA, H., WADA, T., OSAKI, A., YAMADA, H., TOGE, T., NIIMOTO, M. & HATTORI, T. (1990). Relationship between epidermal growth factor receptor status and various prognostic factors in human breast cancer. *Cancer*, **65**, 1980–1984.

VALVERIUS, E.M., VELU, T., SHANKAR, V., CIARDIELLO, F., KIM, N. & SALOMON, D.S. (1990). Over-expression of the epidermal growth factor receptor in human breast cancer cells fails to induce an oestrogen independent phenotype. *Int. J. Cancer*, **46**, 712–718.

WIEBE, V.J., OSBORNE, C.K., MCGUIRE, W.L. & DEGREGORIO, M.W. (1992). Identification of estrogenic tamoxifen metabolite(s) in tamoxifen-resistant human breast tumors. *J. Clin. Oncol.*, **10**, 990–994.

WRIGHT, C., NICHOLSON, S., ANGUS, B., SAINSBURY, J.R.C., FARNDON, J., CAIRNS, J., HARRIS, A.L. & HORNE, C.H.W. (1992). Relationship between c-erbB-2 protein product expression and response to endocrine therapy in advanced breast cancer. *Br. J. Cancer*, **65**, 118–121.

ZUGMAIER, G., ENNIS, B.W., DIESCHAUER, B., KATZ, D., KNABBE, C., WILDING, G., DALY, P., LIPPMAN, M.E. & DICKSON, R.B. (1989). Transforming growth factors type $\beta 1$ and $\beta 2$ are equipotent growth inhibitors of human breast cancer cell lines. *J. Cell. Physiol.*, **141**, 353–361.